# Discrimination between Weaned and Unweaned Atlantic Cod (*Gadus morhua*) in Capture-Based Aquaculture (CBA) by X-Ray Imaging and Radio-Frequency Metal Detector

**DOI:** 10.1371/journal.pone.0095363

**Published:** 2014-04-17

**Authors:** Ekrem Misimi, Svein Martinsen, John Reidar Mathiassen, Ulf Erikson

**Affiliations:** SINTEF Fisheries and Aquaculture, Trondheim, Norway; Louisiana State University School of Veterinary, United States of America

## Abstract

The aim of this study was to investigate the feasibility of two detection methods for use in discrimination and sorting of adult Atlantic cod (about 2 kg) in the small scale capture-based aquaculture (CBA). Presently, there is no established method for discrimination of weaned and unweaned cod in CBA. Generally, 60–70% of the wild-caught cod in the CBA are weaned into commercial dry feed. To increase profitability for the fish farmers, unweaned cod must be separated from the stock, meaning the fish must be sorted into two groups – unweaned and weaned from moist feed. The challenges with handling of large numbers of fish in cages, defined the limits of the applied technology. As a result, a working model was established, focusing on implementing different marking materials added to the fish feed, and different technology for detecting the feed presence in the fish gut. X-ray imaging in two modes (planar and dual energy band) and sensitive radio-frequency metal detection were the detection methods that were chosen for the investigations. Both methods were tested in laboratory conditions using dead fish with marked feed inserted into the gut cavity. In particular, the sensitive radio-frequency metal detection method with carbonyl powder showed very promising results in detection of marked feed. Results show also that Dual energy band X-ray imaging may have potential for prediction of fat content in the feed. Based on the investigations it can be concluded that both X-ray imaging and sensitive radio-frequency metal detector technology have the potential for detecting cod having consumed marked feed. These are all technologies that may be adapted to large scale handling of fish from fish cages. Thus, it may be possible to discriminate between unweaned and weaned cod in a large scale grading situation. Based on the results of this study, a suggestion for evaluation of concept for in-situ sorting system is presented.

## Introduction

The peak season of the commercial cod fisheries in Norway is from January until April, following the migration of mature cod into the Norwegian fjords [5]. During this period, the market supplies of fresh cod increase up to ten times the average yearly supply, whilst in December the market demand for fresh cod exceeds the supply, due to the use of cod as a raw material in Norwegian and European traditional cuisine. The seasonal dependence and variation of the cod fisheries has triggered strategies such as farming of cod, and conservation of the wild caught cod through capture-based aquaculture (CBA). Products deriving from these activities are more suited in terms of volume and quality for the high-demand market in December. Since the dynamics between fisheries, aquaculture and markets is constantly fluctuating, strong year-groups of wild cod have resulted in increased total catch, from 215.000 tons in 2008 to 340.000 tons in 2011. This increase has resulted in diminishing price levels, from 16 NOK (appr. 2 €) per kg round weight (RW) to 11 NOK (appr. 1.50 €) per kg RW in average. This has made commercial cod farmers unable to cope with the lower prices, as the production costs of farmed cod in Norway, normally exceed 18 NOK (2.50 €) per kg RW. The expected production cost from capture based aquaculture might be lower, given efficient conversion of wild caught cod to formulated feed.

The present CBA of cod, with feeding of wild-caught cod, derives from extended trials in the 1980’s [2]. The basic principles of CBA are gentle capture of fish in seine nets and transfer to holding tanks inside fishing vessels. Fish are transferred from the vessels to special flat-bottom cages, specifically developed for storing and resting of cod. Although the operations are performed with caution, the cod can experience physiological changes like gas bladder expansion and severe stress, but the physiological normality is usually established 12 to 24 hours after capture [2].

The captured fish are presented different diets: a) moist feed (frozen herring, mackerel, and capelin); b) semi moist feed (vacuum soaked commercial fish feed, premix of fishmeal and fish oil); and c) dry feed (commercial fish feed). Captured wild cod traditionally prefer moist feed, and complete weaning (adaption to moist feed) will normally occur shortly after introduction [15]. Moist feed consists of frozen blocks of herring and capelin that slowly thaw and disintegrate inside the cage, with the fish foraging upon the thawed feed. Seen from the cod farmer’s point of view, the optimal feed source is commercial dry feed, in terms of nutritionally adequate content, supporting rapid growth, extended storage stability, availability, non-disease carrier and logistics [16]. Fish are reluctant when presented dry feed and the maximum proportion of fully-weaned cod individuals, weaned from moist feed, and varies between 40–70% [16]. Weaned fish achieve satisfying growth, and reportedly increase their weight from 1.5–2 up to 5–6 kg after 5 months. Unweaned fish will not forage on dry feed, resulting in loss of weight and generally poor condition. To achieve viable economy in the cod CBA industry, an efficient method for discriminating unweaned and weaned fish is necessary. Presently, there is no established method for discrimination between weaned and unweaned cod in CBA.

X-ray radiography has been reported as a suitable technique for studying fish feeding, digestion, measuring feed intake and fish growth [14], [8], [9], [10]. In [18], they report use of X-radiography and X-ray dense markers for quantitative determination of gastrointestinal content. [12] used X-ray dense lead glass beads for estimation of individual feed intake of Chinook salmon by X-radiography, while [19] used a similar technique for estimation of feed intake and growth of Atlantic salmon.

The aim of this work was to investigate the required technology platform needed for sorting of wild cod, enabling efficient discrimination between weaned and unweaned cod in CBA. The technological approach involved testing potential methods for in-situ inspection of the gut content after feed intake consisting of commercial dry feed containing markers. Two aspects of the sorting procedure were studied: a) the marking of fish and b) evaluation of detection technology for efficient discrimination. Although the presence of fish feed in fish gut can be possible to detect by use of X-ray images [11], other fish organs such as liver, swim bladder, and gut cavity liquids can disturb the imaging of fish feed. Therefore, marking of fish feed is seen as a potential solution to enhance the detection capability of both X-ray and other detection methods for detection of fish feed.

Two detection technologies that may be suitable for the discrimination between weaned and unweaned cod were evaluated: 1) X-ray imaging in two modes (planar X-ray and dual energy band), with an integrated image analysis, and 2) sensitive radio-frequency metal detection. Five different types of markers were used for discrimination purposes. In addition, the potential of dual energy band X-ray imaging for prediction of fat content in the feed was investigated.

## Materials and Methods

Three experiments were conducted, each investigating a separate detection method for the inspection of gut content to facilitate sorting of weaned and unweaned cod. No human participants were used in the study. The fish was delivered by commercial fishery boats that caught wild cod at the coast of Central Norway. From before, they have all necessary permissions to fish, so no specific permission was needed for this study. Fish was delivered to SINTEF SeaLab packed on ice in styrofoam boxes. The wild cod is not an endangered fish species either in Norway or globally.

### X-ray Imaging of Fish Fed with Commercial Feed with High Contrast Feed Markers

#### Fish

Wild Atlantic cod *(Gadus morhua*) (2.1±0.5 kg, n = 20) were caught at the coast of Central Norway. The fish were killed and packed in ice before being shipped to SINTEF Sealab in Trondheim. Their intestinal content was maintained in the body cavity before proceeding with the investigations.

#### Feed and markers

Fish dry feed [21] was mixed with crude herring oil added as coating for evenly distribution of commercially available markers and to provide a sticky surface of the pellet. The amount of marked feed was 1.0–1.5% of the fish body weight. The markers were chosen so that they were able to provide a good contrast for X-ray image analysis. In addition to metal-based markers shown in Table 1, Ballotini glass beads of 0.6 mm [24] were used at the same dilution ranges (Table 1).

**Table 1:** Dilution ranges for the metal and glass bead markers used in the investigations/experiments.

Marker weight(%)	Marker weight:20 gr feed sample	Marker weight: 30 gr feed sample
0.1	0.02	0.03
0.5	0.1	0.15
1	0.2	0.3
5	1	1.5
10	2	3

The chosen commercially available markers were added to the fish feed at a dilution range from 0.1% to 10% of feed portion weight (Table 1). The size of each feed portion inserted into the fish was between from 20–30 g of fish feed, corresponding to 1–1.5% of fish round weight. This amount corresponds to a typical meal portion size for a large cod [1], [13].

#### Image acquisition and processing

**Figure 1:**
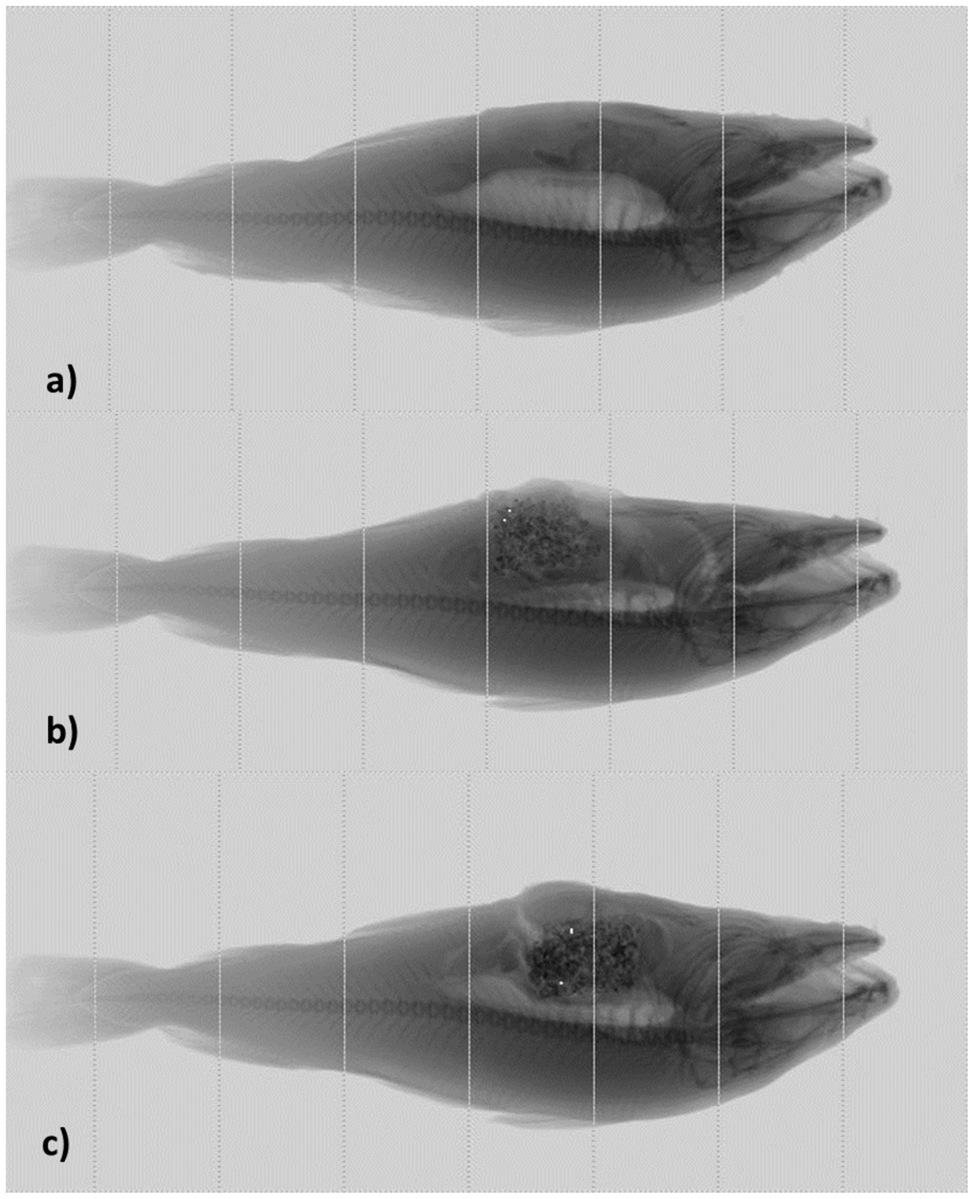
X-ray images of Atlantic cod generated from the Ishida X-ray machine: a) image of a fish without feed, images of a fish with glass beads at b) 0.1% (0.03 g) and c) 10% (3 g) of weight percent of total feed content in gut.

A line scan X-ray setup [7] was used for acquisition of X-ray images. Prior to the experiment, an image acquisition PC was connected to the Ishida apparatus so that the generated images could be saved directly and online into the PC disk. Line scans were stitched in the X-ray machine, resulting in a sequence of 5–6 images of 90×332 pixels per fish while fish was moving on the conveyer belt. These images were then stitched to a full image (Figure 1a) by implementing a stitching function in Matlab R2011b Image Processing Toolbox [23]. The energy level used for acquisition of X-ray images was 60 kV.

### Dual-energy X-ray for Detection of Different Feed Density (Fat Content)

#### Fish

Wild-caught cod (2.5±0.6 kg, n = 10) were killed, bled and chilled in ice. The fish were transported to the Curato X-Ray Clinic in Trondheim. Their intestinal content was maintained in the body cavity while the experiments were carried out. To determine the feasibility, of using dual-energy X-ray imaging for discriminating weaned and unweaned cod, a single cod was used for some preliminary experiments.

#### Feed and markers

Feed samples were weighed and prepared in small plastic bags. Each sample weighed approximately 25 g. The samples were taken from three commercial fish feed diets; Skretting Optiline 2500 (30% fat), Skretting Nutra Parr (22% fat), and BioMar Classic Marine 800 (18% fat). Prior to X-ray imaging, the feed samples were inserted into the fish abdominal cavity through an incision, and the incision was then carefully collated.

#### Image acquisition and processing

**Figure 2:**
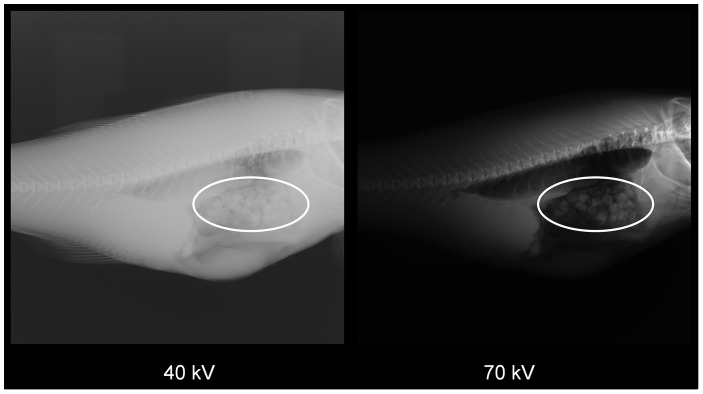
Dual energy X-ray images of Atlantic cod: a) X-ray image at 40 kV, b) X-ray image at 70 kV.

The X-ray images for dual energy X-ray analysis were taken using the commercial medical planar X-ray apparatus [22]. Configuration, setup of parameters, placement and introduction of fish for the imaging system was similar to that described in [20]. To simulate the dual energy X-ray imaging system, fish were imaged with two different X-ray energy levels. A total of 21 planar X-ray images were acquired at both 40 kV (low energy image) and 70 kV (high energy image) (Figure 2). Sets of images of fish without feed, and with NiCr, Steel grit and Cr-Ni alloy markers were generated and saved. The images were transferred then offline into a PC and processed. The Region of Interest (ROI) for dual energy band X-ray image analysis was chosen manually.

These particular voltages were chosen based on the results reported in [20]. Image processing and analysis was done off-line after all images had been acquired. The regions of the images, containing the feed, were segmented from the rest of the fish. Subsequent to this, the regional statistics (consisting on comparing the mean and standard deviation of the image intensity differences between 40 kV and 70 kV images) of the fish regions with feed were compared to those of the same anatomical regions in fish without feed. Features used during the dual energy X-ray image analysis are defined in Table 2, while their mean values are given in Table 3. To test prediction of fat for the fish diets with 18%, 22% and 30% of fat, fat prediction model was developed by using the set of features defined in Table 2 and linear regression. The set of features was extracted from ROI containing fish diets of the abovementioned fat percentages.

**Table 2:** Extracted and generated features used for dual energy X-ray image analysis.

M_40 kV_, S_D40 kV_	Mean Intensity and standard deviation for ROI image at 40 kV
M_70 kV,_ S_D70 kV_	Mean Intensity and standard deviation for ROI image at 70 kV
M_40/70_ = M_40 kV_/M_70 kV_	Ratio between mean intensities of the ROI images at 40 kV and 70 kV
M_40*70_ = M_40 kV_ * M_70 kV_	Multiplication of mean intensities of the ROI images at 40 kV and 70 kV
S_40/70_ = S_40 kV_/S_70 kV_	Ratio between standard deviation for ROI images at 40 and 70 kV
S_40*70_ = S_40 kV_*S_70 kV_	Multiplication of standard deviation for ROI images at 40 and 70 kV

**Table 3:** Mean values of the extracted and generated features from the dual energy X-ay 40 kV and 70 kV ROI images of fish diets of the specific fat percentages (18%, 22%, 30%).

Fat(%)	40 kV mean	40 kV std	70 kV mean	70 kV std	40/70 mean	40/70 std	40*70 mean	40*70 std
18(n = 6)	2587,65	85,70	37,44	7,11	81,71	25,39	91755,42	35862,59
22(n = 6)	2594,17	105,27	40,71	11,06	70,19	17,34	106254,71	32482,88
30(n = 6)	2627,30	134,30	45,67	16,72	65,40	21,80	122088,94	49793,78

### Radio-frequency Metal Detection of Marked Feed in Cod

#### Fish

Wild-caught cod (2.0±0.8 kg, n = 15) caught at the coast of Central Norway, were killed, packed in ice, and shipped to the ACT Group Lab (Jessheim, Norway). As before, the intestines were kept intact during the experiment.

#### Feed and markers

Fish feed from [21] was mixed with four different markers (Table 4), and crude herring oil to get a sticky pellet surface and even distributions of marker particles. A dilution series was created to establish the lower detection limit of each marker. The trial feed with markers were packed into small plastic bags in 20 g portions (approximately 1% of fish body weight), equal to a potential daily feed portion, and inserted within the abdominal cavity of the fish. The abdominal cavity was collated by applying plastic strips around the fish body.

**Table 4:** Particle size of markers used for metal detection.

Marker	Particle size (mm)	Magnetism
Aluminium oxide (AlO2)	0.4–0.6	No
Carbonyl iron (Fe)	0.006–0.009 (powder)	Yes
Steel grit	0.2–0.4	No
Chrome – Nickel alloy (Cr-Ni)	0.6	No

#### Radio-frequency metal detector

**Figure 3:**
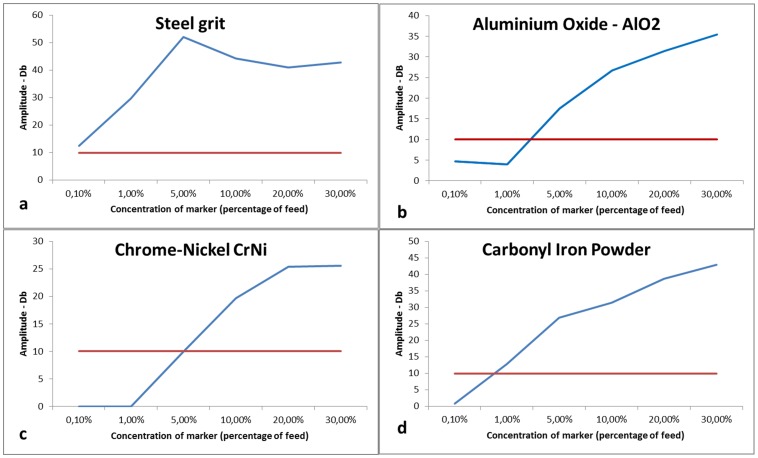
Amplitude of radio frequency metal detector response vs concentration of metal-based markers a) Steel grit, b) Aluminium oxide (AlO_2_), c) Chrome-nickel (CrNi), d) Carbonyl iron powder. The red curve is the detection limit at 10 dB.

The radio-frequency metal detector used for this trial was a CEIA MS21 industrial metal detector [25] supplied by the ACT Group. The detector had a high precision and high sensitivity and was equipped with compensation technology for cancelling external environmental disturbances. The metal detector was arranged over a conveyer belt, approximately 2 m long. The signal strength was given in decibels, and lower detection limit was set to 10 dB (red line in Figure 3). The instrument was calibrated prior to the test by analyzing fish without marked feed and by adjusting the instrument for external disturbance. The metal detector was arranged over a conveyer belt to enable relatively fast and continuous insertion of fish into the detection unit. The sampling/acquisition period per fish was approximately 2 sec, but it can potentially be decreased by optimizing the system setup.

### Statistical Analysis

To develop the linear regression model for fat prediction of fish diets inserted in the fish gut cavity, the Minitab [26] statistical software was used. The image features, mean and standard deviation of the X-ray image intensity (Table 2, 3), extracted from dual energy X-ray images, were fed into the Minitab and fat prediction models were generated. The combinations used, in form of multiplication and ratio of features, are motivated from the fundamental principle in dual energy X-ray imaging where the high energy and low energy images are combined in multiplication and division image processing operations [4].

## Results and Discussion

### X-ray

**Figure 4:**
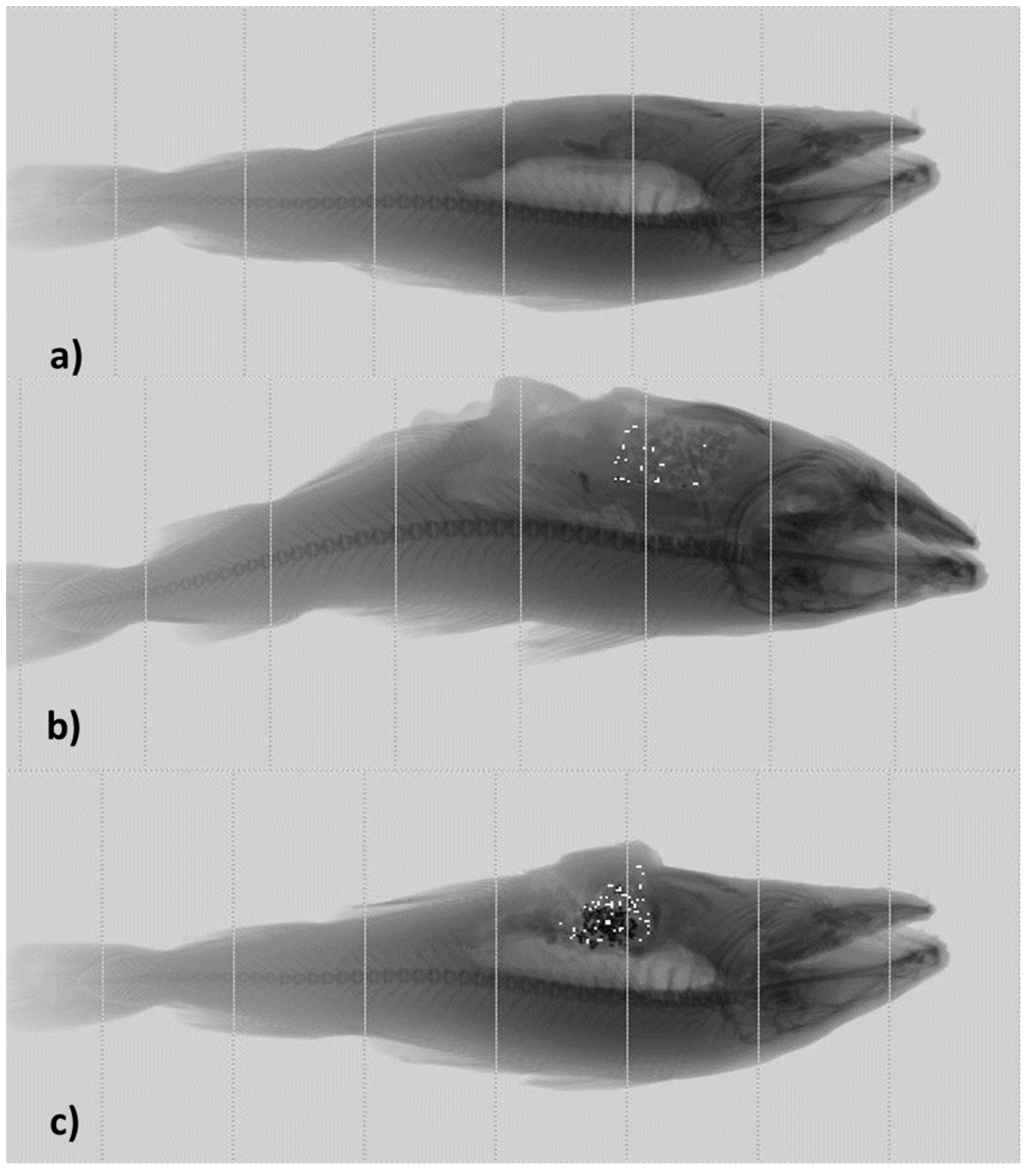
X-ray images of Atlantic cod generated from the Ishida X-ray machine: a) image of fish without feed, b) image of fish with Chrome-Nickel (CrNi) markers at 0.1% (0.03 g) weight percent of total feed content in gut; c) image of fish with Chrome-Nickel (CrNi) markers at 10% (3 g) weight percent of total feed content in gut.

In Figure 1 and 4 are shown the X-ray images of fish without feed versus fish with feed mixed with glass beads (Figure 1b, 1c) and metal-based markers (Figure 4b, 4c) at the different dilution ranges (Table 1). Figure 1a shows a good visibility of the swim bladder. In Figure 1b and 1c there are also a good visibility of fish feed although its segmentation was not straightforward. The white dots were a result of automatic detection and segmentation of glass beads by the Ishida X-ray machine at a certain threshold. It is seen that increase in concentration of glass markers did not result in a better discrimination (Figure 1c).

In Figure 4b and 4c are shown the X-ray images of cod with feed mixed with different concentrations of metal markers. Figure 4b shows a fish with feed containing 0.1% marker (0.03 g), while Figure 4c shows a fish with feed containing 10% marker (3 g). As it is seen in the Figure 4c, the image segmentation of particles was effective at the 10% marker concentration. This is in line with the conclusions of the preliminary study [11], who suggest that the feed could be added particles that absorb X-ray to increase contrast and thereby enable the discrimination of cod weaned to feed.

The advantage of X-ray as a non-invasive method for sorting and discrimination ability at the 10% particle metal concentration (3 g) is shown. Some potential challenges for the methods could be: 1) the need for metal particles to be mixed into the fish feed; 2) to achieve the necessary accuracy, each fish must be singulated prior to imaging. The singulation of fish can be technically solved by use of commercially available singulators. Although prospects of sorting large amounts of fish from a cage containing typically 50.000–100.000 individuals may seem challenging for a concept based on X-ray imaging, there already are commercially available X-ray imaging systems that can cope with harsh environment such as X-ray imaging systems for reservoir studies or pipes provided by InnospeXion [6].

### Dual energy X-ray

An example of the visibility of undigested feed [21], with 30% fat, is seen in Figure 2, showing that the feed is visible in both the 40 kV and 70 kV images. Despite the visibility of the feed structure, in the form of cuboid chunks, we conclude that the individual variations in cod thickness, and variations in feed spatial distribution within the gut, makes planar dual-energy X-ray imaging unsuited for the purpose of measuring feed content in the gut. However, the method is promising for measurement of fat percentage in the fish feed diets, assuming the precise extraction of the ROI to be analyzed. Depending on the set of features, the prediction of fat from both linear and multiple linear regression varies from poor R^2^ = 0,4476 (use of only one feature M_40*70_ = M_40 kV_ * M_70 kV_ ) to R^2^ = 0,9029 (use of all generated features) (Table 5). To obtain fat percentage measurements with higher accuracy and throughout the fish gut volume, a dual-energy computed tomography (CT) imaging approach may be convenient, as indicated in previous research [17].

**Table 5:** Prediction of fat of fish diets inserted into fish gut cavity with dual energy band X-ray image features.

Feature set	Prediction model	R^2^
M_40*70_ = M_40 kV_ * M_70 kV_	Fat(%) = 2,42+0,000196*M_40*70_	0,4476
All features	Fat(%) = 517+0,00005* 40*70 M −0,203*40 kVmean −0,383 * 40 kV std +0,97* 70 kVmean−15,1 *70 kV std −0,12* 40/70 mean +1,29* 40/70 std +0,00566* 40*70 std	0,9029

### Radio-frequency Metal Detector

The radio-frequency metal detector gave consistent measurements (Table 6). All markers gave detection responses above the detection limit for metal concentrations between 0.1 and 5%. Steel grit marker at 0.1% (0.03 g) concentration provided the strongest signals, while carbonyl iron powder did so at approximately 0.7% (2.1 g) (Figure 3). The steel grit particles were relatively large, with sharp edges and not of uniform shape. The carbonyl-iron powder could be an excellent marker due to its low particle size (a few µm), round particle shape, adaptability to processing in commercial feed mill technology, and benign interaction with the cod digestive system.

**Table 6:** Response values from the metal detector for different marker types and concentrations (detection limit at 10 dB).

Concentration (%)	AlO_2_(dB)	CrNi(dB)	Steel grit(dB)	Carbonyl Iron powder(dB)
0.10	4.62	0	12.42	0.9
1.00	3.9	0	29.68	**12.84**
5.00	17.52	9.96	52.02	**26.9**
10.00	26.74	19.7	44.22	**31.36**
20.00	31.48	25.34	41	**38.64**
30.00	35.46	25.54	42.78	**42.86**

It is seen that the response value for Carbonyl Iron powder is above the limit for concentrations higher than 1%.

Due to efficiency and detection accuracy, radio-frequency metal detectors combined with feed-added carbonyl iron powder at low concentrations seemed like a robust and efficient system for detection of feed in an *in vivo* context. The radio-frequency metal detector could easily be coupled with a conveyer belt or a pipe system where fish can be inspected and sorted regardless of positioning and speed.

### Use of Fish Feed and Markers

The use of fish feed with added markers (NiCr, AlO_2_, Carbonyl Iron, Steel Grit, Ballotini glass beads), as opposed to other marking methods, provided an effective and low-cost method for marking of fish. The general advantage of using this method was that the marked feed could be manufactured in a commercial feed extruder, and the feed could be distributed to all fish in the cohort at once. One potential disadvantage be the variable marking efficiency when feed is unevenly distributed or not accepted by the fish.

### Biological Hazards and Fish Welfare

Since we worked with dead fish under laboratory conditions, possible biological hazards and animal welfare issues related to the application of the different markers were not tested in this study.

In practice with live fish, however, it is hardly conceivable that markers such as steel grit, aluminum oxide and Cr-Ni alloys are suitable in fish feed since these compounds are either abrasive, they can have sharp edges, or they can be carcinogen to humans. As such, these compounds might harm the digestive system in fish and their use can therefore result in poor fish welfare. If absorbed in the flesh, they might constitute a hazard to the consumers. Although the abovementioned markers don’t have a foreseeable practical use in live fish, we used them for method development as they were commercially available. One of the main hypothesis whether the use of markers would increase the contrast in detection of marked feed to be able to discriminate cod weaned to feed.

On the other hand, results imply that carbonyl iron has an excellent foreseeable practical use in live fish due to the fact that it does not pose biological hazard to live fish. In human beings, single doses of 1–10 g carbonyl iron were tolerated with no evidence of toxicity and only minor gastrointestinal side effects [3]. Future work will be concentrated on developing trials with live fish and using carbonyl iron powder as feed marker.

### Concept for Implementation of Sorting System

**Figure 5:**
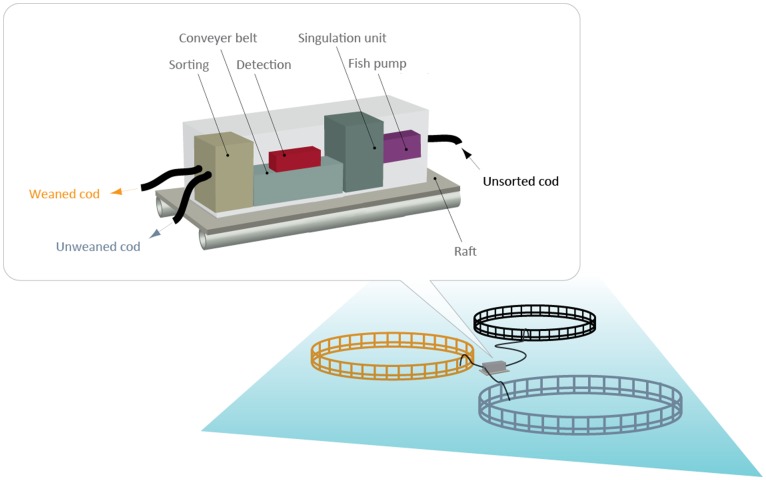
Principle diagram of the concept of sorting system for in situ discrimination of weaned and unweaned Atlantic cod in capture-based aquaculture (CBA) systems.

With a detection/discrimination subsystem based on either X-ray imaging or radio frequency metal detection, a suggested concept for implementation of a sorting system for discriminating between weaned and unweaned cod is shown (Figure 5). The discrimination process begins by feeding all the cod with commercial dry feed mixed with a small concentration of carbonyl iron powder. Once the feeding ceases, the cod are pumped from the cage into the sorting system. By mounting a fish pump, detection and sorting unit on a raft, the fish farmer could cut the costs by integrating the discrimination subsystem into the existing pump and sorting system. After drain-off of water, a conveyer belt can be used to transport the fish to the X-ray imaging system or the radio-frequency metal detection unit. Based on the result from the detection unit, with weaned cod being detected by the presence of the metallic carbonyl iron powder in the ingested dry feed, the weaned and unweaned cod can be transported to separate cages. From this point on, weaned cod can thus be fed with commercial dry feed, whereas the unweaned cod are fed with the more costly moist feed.

An evaluation of investment costs for the concept solutions based on X-ray, dual energy X-ray and metal detector discrimination methods is reported in [16]. This analysis takes into account the investment need for pumping system, singulation unit, raft, flexible pipes, detection unit, conveyer belts, control software, housing, and costs related to feed production. The cheapest concept solution is the one based on metal detector concept with an estimated cost of 43806 € (361000 NOK), while the concepts based on X-ray are estimated at an approximate cost of 62918 € (518500 NOK).

## Conclusions and Future Work

The aim of this work was to investigate the feasibility of X-ray imaging and radio frequency metal detection methods for use in discrimination and sorting of wild cod into two groups – those that are unweaned and those that are weaned from moist feed. Based on the current research, we conclude that both X-ray imaging and radio frequency metal detection were promising methods for discrimination between unweaned and weaned wild cod. In the present investigation, the best results were obtained with the method based on the use of carbonyl iron powder in the commercial dry fish feed combined with the high sensitive radio-frequency metal detector technology. Metal detector based concept was also better when it comes to investment cost, capacity, accuracy, and use in water. Therefore, as future work we suggest building of a prototype based on metal detector concept, and evaluation of the concept for sorting of live fish in the controlled laboratory conditions. Another important aspect of these trials would be to document the potential biological hazards of carbonyl iron powder to live fish.
